# Interlinking FinTech and eHealth: a qualitative study

**DOI:** 10.3389/fpubh.2024.1398136

**Published:** 2024-08-01

**Authors:** Fahad Al-Anezi

**Affiliations:** Department Management Information Systems, Imam Abdulrahman Bin Faisal University, Dammam, Saudi Arabia

**Keywords:** FinTech, eHealth, Saudi Arabia, interlink, benefits, healthcare, digital payments

## Abstract

**Introduction:**

This study investigates the integration of financial technology (FinTech) and electronic health (eHealth) to explore the opportunities, challenges, and implications arising from their interlinkage in Saudi Arabia.

**Methods:**

Utilizing qualitative semi-structured interviews with 26 participants—including physicians, patients, technical and administrative managers, and FinTech consultants—the research adopts an inductive approach to understand diverse perspectives.

**Results:**

Key findings reveal significant benefits such as improved efficiency in administrative processes, enhanced access to healthcare services, increased financial inclusion, better decision-making, improved patient experience, and the promotion of innovation and sustainability. However, barriers including regulatory challenges, data privacy and security concerns, interoperability issues, the digital divide, resistance to change, and cost implications were also identified.

**Conclusion:**

Overall, the integration of FinTech and eHealth holds substantial promise for advancing healthcare delivery in Saudi Arabia. Future implications include the expansion of telehealth services, an increase in startups, the integration of wearable health devices, blockchain-based systems, evolving regulatory frameworks, and heightened collaborations. Addressing the identified challenges is crucial for realizing the full potential of this integration.

## 1 Introduction

Financial technology, commonly known as FinTech, refers to the innovative use of technology to deliver financial services and products in a more efficient, accessible, and user-friendly manner (1). FinTech encompasses a wide range of applications, including mobile banking, peer-to-peer lending, digital payments, blockchain technology, robo-advisors, and crowdfunding platforms, among others. By leveraging advanced algorithms, data analytics, and digital interfaces, FinTech companies aim to streamline traditional financial processes, enhance financial inclusion, and democratize access to financial services (2). FinTech has transformed the financial landscape, empowering individuals, businesses, and institutions to manage their finances, make transactions, and invest with greater convenience, speed, and transparency than ever before (3).

Electronic health (eHealth), refers to the integration of digital technologies into healthcare systems to improve the delivery, accessibility, and quality of healthcare services. This includes a wide range of applications such as electronic health records (EHRs), telemedicine, wearable health devices, health apps, and remote patient monitoring systems (4). By leveraging digital platforms, data analytics, and communication technologies, eHealth initiatives aim to enhance patient care, optimize healthcare workflows, and empower individuals to take a more proactive role in managing their health (5).

FinTech and eHealth are two areas that have received a significant amount of attention in the current environmental landscape of technological growth due to the revolutionary potential that they possess. Traditional financial systems can be completely transformed by FinTech, which is characterized by the provision of novel financial services that are made possible by technology (1–3). At the same time, eHealth, which refers to the use of digital technologies into healthcare, has made the promise of enhancing patient care, streamlining processes, and improving overall health outcomes. A growing number of people are becoming aware of the possible synergies that could result from the confluence of these two industries, despite the fact that these fields have traditionally been investigated independently (6).

Studying the integration of FinTech and eHealth from the viewpoints of experts in both domains is crucial for a comprehensive understanding of the opportunities and challenges therein. eHealthcare experts offer insights into healthcare systems’ intricacies, regulatory frameworks, and clinical standards, while FinTech experts provide expertise in digital finance, payment systems, and regulatory compliance. Collaboration between these experts facilitates innovative thinking and the identification of synergies, enabling the discovery of novel avenues for improving healthcare services and reducing costs. Furthermore, these perspectives help anticipate and address challenges such as data security concerns and interoperability issues, driving evidence-based policymaking and innovation in both sectors (7–9). Ultimately, this interdisciplinary approach aims to enhance patient care delivery and outcomes through the effective integration of financial technology into healthcare systems.

FinTech and eHealth are two areas that have garnered significant attention in the current landscape of technological growth due to their revolutionary potential. While FinTech can transform traditional financial systems by providing novel financial services enabled by technology, eHealth promises to enhance patient care, streamline processes, and improve overall health outcomes through digital technologies. Despite these fields traditionally being investigated independently, a growing number of people are recognizing the potential synergies from their confluence. This study aims to explore and understand the complex interplay between FinTech and eHealth through a qualitative research framework. The specific research questions guiding this study are:

1. What opportunities arise from the integration of FinTech and eHealth in improving healthcare delivery and patient welfare?
2. What challenges and barriers must be addressed to facilitate the effective integration of FinTech and eHealth?
3. How do key stakeholders, including healthcare professionals, FinTech consultants, and patients, perceive the interlinking of FinTech and eHealth?

By engaging diverse stakeholders through qualitative semi-structured interviews, this research seeks to interpret the perspectives and experiences of participants regarding the interlinking of FinTech and eHealth. The goal is to provide insights into how FinTech can be effectively leveraged to strengthen eHealth initiatives, thereby contributing to the progress of healthcare delivery and patient welfare in Saudi Arabia.

## 2 Background study

The interlinking of FinTech and eHealth signifies the convergence of two innovative sectors poised to revolutionize conventional frameworks. FinTech, encompassing a wide range of financial services utilizing technology, has experienced tremendous global expansion and acceptance (10–14). Innovations such as mobile banking and blockchain-powered transactions have improved financial accessibility and transformed how individuals and organizations engage with money (15, 16). Similarly, advancements in digital technology have driven eHealth projects to tackle persistent difficulties in healthcare delivery. Innovations such as telemedicine, electronic health records (EHRs), wearable health devices, and AI-driven diagnostics are revolutionizing healthcare systems globally (17–21).

The integration of FinTech with eHealth holds great potential for addressing critical issues in healthcare systems. For instance, streamlining healthcare payments and insurance procedures through FinTech technologies like mobile payment platforms and blockchain-based smart contracts can simplify invoicing, claims processing, and reimbursement processes, leading to lower administrative costs and improved financial visibility (9). Additionally, implementing digital payment methods can enhance financial inclusion for marginalized groups, ensuring equitable access to healthcare services (22–24).

Globally, there are successful examples of FinTech-eHealth integrations. For instance, in Kenya, the mobile payment platform M-Pesa has been used to facilitate healthcare payments, allowing users to pay for medical services and insurance premiums easily (25–27). This has significantly increased healthcare accessibility for underserved populations. In Estonia, the implementation of blockchain technology in eHealth records has enhanced data security and interoperability, enabling seamless and secure access to patient information across different healthcare providers (28–30). These examples highlight the potential benefits of integrating FinTech and eHealth, providing a roadmap for similar initiatives in other regions.

However, integrating FinTech and eHealth also poses significant challenges. Data security and privacy are major concerns (31), as both sectors handle sensitive personal information. Ensuring robust cybersecurity measures and adherence to data protection regulations is paramount. Interoperability issues between different FinTech and eHealth platforms can hinder seamless integration, necessitating the development of standardization and interoperability frameworks. The digital divide presents another barrier, as discrepancies in digital literacy, internet access, and the availability of smartphones or laptops can exacerbate existing health disparities, particularly among vulnerable populations (32–37).

The integration of FinTech with eHealth has significant consequences for politicians, healthcare providers, and technology developers. Policymakers need to manage regulations to provide a supportive climate for innovation while protecting consumer rights and privacy. It is crucial to establish collaborative relationships among public health agencies, financial regulators, and technology companies to create regulatory frameworks that support interoperability, data security, and consumer protection (38, 39). Healthcare providers can get advantages from integrated FinTech-eHealth solutions by improving operational efficiency, enhancing patient engagement, and achieving better clinical outcomes. Healthcare businesses can optimize their operations, lessen administrative tasks, and enhance healthcare accessibility by using digital payment methods, automated billing systems, and telemedicine platforms (40–42). Healthcare providers must prioritize the ethical use of patient data and uphold trust in digital health services. Technology developers are crucial in fostering innovation where FinTech and eHealth meet. Developers can create customized solutions to tackle healthcare difficulties by working with healthcare stakeholders and utilizing advanced technologies like artificial intelligence (AI) and machine learning (ML). User-centric design concepts and a focus on inclusivity are crucial to guarantee that FinTech-eHealth solutions are accessible and fair for all persons, regardless of their socioeconomic background or technological ability.

Despite the growing interest in FinTech and eHealth independently, there is a notable gap in the qualitative exploration of their integration. Existing studies (24–42) often focus on the technical and operational aspects of these fields, with limited attention to the nuanced, contextual understanding of how they intersect. This study aims to fill this gap by examining the perspectives and experiences of key stakeholders involved in both domains, providing a deeper, qualitative insight into the opportunities and challenges of their interlinkage.

This study’s contribution lies in its qualitative approach to exploring the interplay between FinTech and eHealth. By engaging diverse stakeholders, including healthcare professionals, FinTech consultants, and patients, the research aims to uncover the lived experiences, perceptions, and challenges faced in the integration process. This approach provides a richer, more contextual understanding of the factors influencing the successful interlinkage of these two domains.

In summary, while there is a growing recognition of the potential synergies between FinTech and eHealth, there remains a significant gap in qualitative research exploring this intersection. This study seeks to address this gap by providing in-depth insights into the opportunities and challenges of integrating FinTech with eHealth, thereby contributing to the broader discourse on innovative solutions for healthcare delivery and patient welfare.

## 3 Methods

### 3.1 Study settings and participants

This study utilized a qualitative research design to explore the integration of FinTech and eHealth. A qualitative approach was chosen because it allows for an in-depth understanding of participants’ experiences, perceptions, and the complex interplay between these domains. Unlike quantitative or mixed methods, a qualitative study is particularly suited for exploring nuanced, context-specific phenomena and generating rich, detailed insights that quantitative methods may not capture. Both purposive and convenience sampling strategies were utilized in this investigation, as is typical for similar studies (43). The participants consisted of healthcare professionals such as physicians specialized in eHealth, hospital managers with administrative and technical roles from a public and a private hospital, FinTech specialists acting as advisors, and patients utilizing eHealth technologies. All participants were adults over 18 years old living in Saudi Arabia, encompassing both males and females. The selection process involved identifying key informants within healthcare institutions and FinTech firms, as well as reaching out to patients who had experience with eHealth services. The purposive sampling ensured that participants had relevant knowledge and experience, while convenience sampling facilitated practical access to these individuals.

### 3.2 Interview guide development

A detailed interview guide was developed based on a comprehensive review of existing literature (9, 22–24, 31–42) and aligned with the study’s objectives. The guide consisted of nine main inquiries designed to elicit participants’ views on the integration of FinTech and eHealth, including potential benefits, challenges, and future implications. Follow-up questions such as “What do you mean?” and “Can you clarify, please?” were included to prompt deeper exploration and detailed responses. The interview guide was piloted with a small group of participants to ensure clarity and relevance. Feedback from the pilot was used to refine the questions and ensure they effectively captured the participants’ perspectives.

### 3.3 Rationale for methodological choices

The qualitative approach was chosen over quantitative or mixed methods because it supports the study’s objective of exploring the complex and context-specific interplay between FinTech and eHealth. Qualitative methods are particularly effective in capturing the depth and diversity of participants’ experiences and perceptions, providing rich, detailed insights that are essential for understanding the integration of these two domains. Semi-structured interviews were chosen for this study because of their flexibility in data collection, which enables a balance between standardized questions and open-ended inquiry. This is essential for investigating varied perspectives from different stakeholders in eHealth and FinTech in Saudi Arabia. Open-ended interviews allow for capturing a wide range of perspectives, increasing participant involvement and trust, and supporting continuous data collection and analysis, which are crucial for revealing new insights and comprehending intricate phenomena in a particular context. This method is in line with the complex nature of the research subject and the varied backgrounds of participants, thereby enhancing the credibility and dependability of the study results. When conducting semi-structured interviews, it is important to address ethical difficulties by considering various factors. Initially, participants were provided with informed consent to ensure they comprehended the study’s purpose, their voluntary involvement, and the confidentiality of their input. Autonomy and confidentiality were upheld during the discussions, with precise instructions given on how sensitive information would be managed and shared (44). By engaging diverse stakeholders through semi-structured interviews, the study aimed to uncover the lived experiences and nuanced perspectives that quantitative methods might overlook. Thematic analysis enabled a thorough examination of the data, allowing for the identification of significant patterns and themes that contribute to a comprehensive understanding of the research topic.

### 3.4 Data collection

The interviewees were invited to participate, and a schedule was arranged upon getting their approvals via email. Interviews were conducted via the Zoom application online. The interviews were conducted in both Arabic and English based on the participants’ choices due to the widespread use of Arabic in Saudi Arabia. Comprehensive information was gathered from the interviews by recording the sessions. Each session lasted around 60 min. Interviews were carried out over a four-week period with a total of 26 participants, including five physicians, three technical managers, two administrative managers, seven FinTech consultants, and nine patients recruited from the two hospitals previously stated.

### 3.5 Data analysis

The audio data was transcribed into text for digital storage and organization. The Arabic material was thereafter translated into English by two experienced translators. The translations were cross-checked and proofread by authors to ensure the exact meaning was preserved in all sentences, enabling the effective utilization of qualitative analytic methods. A thematic analysis approach was employed to analyze the data. This involved a systematic process of coding the data, identifying patterns, and developing themes. The analysis followed Braun and Clarke’s (45) six-phase framework: familiarizing with the data, generating initial codes, searching for themes, reviewing themes, defining and naming themes, and producing the report. MAXQDA 2022 software was used to facilitate the organization and categorization of the data.

### 3.6 Ethical considerations

The study received ethical approval from the institutional review board of Imam Abdulrahman Bin Faisal University in Saudi Arabia. Participants were informed about the study’s purpose, their voluntary involvement, and the confidentiality of their input. Pseudonyms and codes were used to ensure the anonymity of participants, and all data was securely stored and managed.

## 4 Results

From the thematic analysis, a total of 91 codes were identified and categorized into four main themes: Benefits, Barriers, Overall Impression, and Future Implications. Each theme was further divided into sub-themes, providing a comprehensive understanding of the integration of FinTech and eHealth.

### 4.1 Benefits

#### 4.1.1 Improved efficiency

Participants highlighted that integrating FinTech and eHealth streamlines administrative processes such as billing, claims processing, and payment transactions, reducing paperwork and manual errors. This efficiency translates to cost savings for healthcare providers and insurers, allowing resources to be reallocated toward improving patient care and expanding healthcare services. For instance, an administrative manager noted, “The integration of digital payment systems has drastically reduced the time and effort we spend on processing insurance claims.”

#### 4.1.2 Improved access to healthcare services

Many participants, especially patients, observed that FinTech solutions enable them to access services such as online appointment booking, telemedicine consultations, and remote monitoring, making healthcare more accessible, particularly in remote or underserved areas. One patient remarked, “Booking an appointment and having a telemedicine consultation from my village has been a game-changer. I do not have to travel long distances to see a doctor.”

#### 4.1.3 Financial inclusion

FinTech initiatives promote financial inclusion by offering digital payment solutions, micro-insurance, and healthcare financing options tailored to individual needs. By providing convenient and affordable payment methods, FinTech enables individuals to access healthcare services without the financial burden of upfront costs. A physician stated, “The new government rules mandate health insurance for all employees, and FinTech ensures timely payments, ensuring everyone has access to necessary healthcare services.”

#### 4.1.4 Improved decision-making

Participants, particularly administrative and technical managers, observed that FinTech integration enables the collection, analysis, and utilization of healthcare data to inform decision-making processes. Leveraging advanced analytics and artificial intelligence, healthcare providers can gain insights into patient preferences, healthcare utilization patterns, and population health trends. An administrative manager mentioned, “We are now able to analyze data trends to better allocate resources and meet the growing demand for remote consultations.”

#### 4.1.5 Improved patient experience

The seamless integration of eHealth and FinTech solutions enhances the patient experience by offering convenient digital platforms for managing healthcare appointments, accessing medical records, and communicating with healthcare providers. One patient shared, “I can now book an appointment, pay online, and get my lab results from home. This convenience has improved my overall experience with the healthcare system.”

#### 4.1.6 Promotion of innovation and sustainability

Few FinTech consultants noted that entrepreneurs in the FinTech space are collaborating with healthcare providers to create novel solutions for addressing healthcare challenges, driving continuous improvement and advancement in the Saudi healthcare ecosystem. A FinTech consultant commented, “The push for innovation is aligned with Saudi Vision 2030, aiming for a sustainable and fully digitized healthcare system.”

### 4.2 Barriers

#### 4.2.1 Challenges in regulations

Most FinTech consultants observed that regulatory frameworks and lack of clear guidelines may impede the adoption of FinTech solutions in healthcare. Navigating new regulatory requirements specific to FinTech integration poses a significant challenge for startups and healthcare institutions. One consultant remarked, “The regulatory environment is complex, and it often takes time to ensure compliance with both financial and healthcare laws.”

#### 4.2.2 Data privacy and security

Participants raised concerns over data breaches, cyberattacks, and unauthorized access to personal health information, which may hinder the adoption of eHealth and FinTech technologies. A technical manager highlighted, “Ensuring robust cybersecurity measures is crucial as we deal with highly sensitive patient data.”

#### 4.2.3 Interoperability

Interoperability challenges between FinTech platforms and existing healthcare systems were highlighted as significant hurdles. Integrating different EHR systems with various billing and payment applications is complex. A technical manager explained, “Different systems often do not communicate well, making seamless integration difficult.”

#### 4.2.4 Digital divide

Several participants, including physicians, patients, and FinTech consultants, noted that limited digital literacy and uneven access to technology among certain population segments exacerbate health disparities and hinder the adoption of FinTech-enabled healthcare services. A physician stated, “Elderly patients and those in rural areas often struggle with using new technologies, which limits their access to digital health services.”

#### 4.2.5 Resistance to change

Some healthcare professionals and patients exhibit resistance to adopting new technologies and digital workflows, preferring traditional methods of healthcare delivery. A FinTech consultant observed, “Change management and user education are essential to demonstrate the benefits of FinTech-enabled healthcare services.”

#### 4.2.6 Cost implications

While FinTech solutions can improve efficiency and reduce healthcare costs in the long run, initial investment costs for implementing and integrating new technologies may be prohibitive for some healthcare providers. An administrative manager noted, “Smaller clinics and rural hospitals often lack the financial resources for significant technology investments.”

### 4.3 Overall impression

The overall impact of FinTech integration on healthcare services is overwhelmingly positive, marked by improvements in efficiency, accessibility, affordability, and patient experience. For example, an administrative manager highlighted, “FinTech solutions have significantly reduced our operational costs, allowing us to allocate more resources toward patient care.” Participants also emphasized the need for addressing identified barriers to maximize the benefits of FinTech in healthcare. One physician stated, “To fully realize the potential of FinTech and eHealth integration, we need to focus on regulatory frameworks, cybersecurity, and bridging the digital divide.”

### 4.4 Future implications

#### 4.4.1 Expansion of telehealth services

Physicians observed a rapid increase in the use of telehealth services and opined that FinTech solutions will play a crucial role in facilitating telehealth transactions, including online consultations, remote patient monitoring, and electronic prescriptions. A physician mentioned, “Telehealth has become an essential part of our practice, and FinTech makes it easier to manage these services efficiently.”

#### 4.4.2 Increase in startups and entrepreneurs

With increased support from the government, especially for innovators as part of Saudi Vision 2030, there is a growing number of Healthtech startups focusing on developing FinTech-enabled solutions to address specific healthcare challenges. A FinTech consultant noted, “We are seeing a surge in startups developing innovative solutions to bridge gaps in healthcare delivery.”

#### 4.4.3 Integration of wearable health devices

The proliferation of wearable health devices such as fitness trackers, smartwatches, and medical-grade sensors presents opportunities for leveraging FinTech to integrate these devices into the healthcare ecosystem, promoting IoT adoption. A technical manager explained, “Integrating wearable devices with FinTech solutions can provide continuous health monitoring and better patient care.”

#### 4.4.4 Blockchain-based healthcare systems

Blockchain holds promise for enhancing data security, interoperability, and transparency in healthcare systems. A FinTech consultant observed, “Blockchain technology can revolutionize how we manage and secure health data, making it more accessible and reliable.”

#### 4.4.5 Evolution in regulatory frameworks

As demand for FinTech adoption in healthcare grows, Saudi regulators may introduce new regulations or guidelines to address emerging challenges related to data privacy, cybersecurity, interoperability, and consumer protection while fostering innovation and competition. An administrative manager remarked, “Evolving regulatory frameworks are essential to support the integration of new technologies in healthcare.”

#### 4.4.6 Increased partnerships and collaborations

Collaboration between healthcare providers, FinTech companies, government agencies, and other stakeholders is expected to increase, driving co-creation and implementation of innovative FinTech-enabled healthcare solutions. A FinTech consultant projected, “Collaborative efforts are crucial for developing and implementing solutions that enhance healthcare delivery.”

## 5 Discussion

This study explored the perspectives of various stakeholders, including healthcare professionals, FinTech consultants, and patients, to understand the opportunities, challenges, and implications of integrating FinTech and eHealth technologies in the healthcare sector.

One of the key findings of the study is the overwhelmingly positive impact of FinTech integration on the quality of healthcare services in Saudi Arabia. The integration of Fintech solutions, such as digital payments, telemedicine, and wearable health devices, has led to improvements in efficiency, accessibility, affordability, and patient experience. The FinTech transaction value in Saudi Arabia was identified to be $56.29 billion in 2023, which is projected to reach $87.14 billion in 2028, with 36.71 million users in Saudi Arabia (46). A CAGR of 12.14% is forecasted between 2024 and 2029 in relation to Saudi FinTech market (47), compared to 14% CAGR globally (48). Furthermore, the digital payments market transaction value in Saudi Arabia is projected at $63.9 billion ($11.55 trillion – worldwide) in 2024 and would reach $87.14 billion ($16.62 trillion – worldwide) in 2028, reflecting a CAGR of 8.06% (9.52% worldwide) (49, 50). These benefits align with global trends in FinTech and eHealth, where digital innovations are reshaping traditional healthcare delivery models and empowering individuals to take a more proactive role in managing their health. Furthermore, as observed in (22–24, 31–35) the findings in this study highlighted the important aspect of increasing healthcare accessibility to vulnerable groups by integrating FinTech services into eHealth.

However, despite the potential benefits, the study also identified several barriers to FinTech integration in healthcare. Regulatory challenges, data privacy and security concerns, interoperability issues, digital divide, resistance to change, and cost implications were among the prominent barriers highlighted by participants in similar to the previous studies (38–42, 51, 52). These barriers underscore the complexities and challenges associated with integrating FinTech and eHealth technologies into the Saudi healthcare system, requiring careful consideration and strategic planning by policymakers, regulators, healthcare providers, and technology developers.

To address these barriers and maximize the benefits of FinTech integration in healthcare, the study suggests several measures. These include updating regulatory frameworks, investing in cybersecurity infrastructure, and training programs, promoting digital literacy and internet connectivity, fostering collaboration between stakeholders, and raising public awareness about the benefits of FinTech integration (53–55). These measures are essential for creating an enabling environment for innovation and ensuring the successful implementation of FinTech-enabled healthcare solutions in Saudi Arabia.

### 5.1 Implications

Looking ahead, the study identifies future trends and implications of interlinking FinTech and eHealth in Saudi Arabia. These include the expansion of telehealth services, growth in Healthtech startups, integration of wearable health devices, adoption of blockchain-based healthcare systems, evolution in regulatory frameworks, increased partnerships and collaborations, and emphasis on sustainable and inclusive healthcare solutions (15–21). These trends highlight the potential for continued innovation and transformation in the Saudi healthcare ecosystem, driven by the convergence of FinTech and eHealth technologies.

Theoretical implications of this study underscore the importance of interdisciplinary research in exploring the convergence of FinTech and eHealth, shedding light on the complex dynamics and potential synergies between these two domains. By adopting a qualitative research approach and engaging diverse stakeholders, this study contributes to theoretical frameworks that elucidate the multifaceted impacts of FinTech integration on healthcare delivery and patient outcomes. Moreover, the study’s findings offer theoretical insights into the regulatory, technological, and socio-economic factors shaping the adoption and diffusion of FinTech-enabled healthcare solutions in the context of Saudi Arabia, enriching scholarly discourse on the intersection of finance, technology, and healthcare.

Practical implications of this study are manifold and extend to policymakers, regulators, healthcare providers, technology developers, and other stakeholders involved in shaping the future of healthcare delivery in Saudi Arabia. The study’s findings provide practical guidance for policymakers and regulators in designing supportive regulatory frameworks that facilitate the safe and effective integration of FinTech and eHealth technologies. Healthcare providers can leverage the insights from the study to inform strategic planning and decision-making, enabling them to harness FinTech solutions to improve operational efficiency, enhance patient engagement, and deliver more personalized and accessible healthcare services. Likewise, technology developers can draw upon the study’s findings to design and deploy innovative FinTech-enabled healthcare solutions that address the identified barriers and capitalize on the opportunities identified by stakeholders. Overall, the study’s practical implications underscore the transformative potential of FinTech integration in advancing healthcare delivery and patient outcomes in Saudi Arabia, offering actionable insights for driving positive change in the healthcare ecosystem.

### 5.2 Recommendations

Based on the findings of this study, several recommendations can be proposed for Saudi Arabian healthcare institutions and policymakers to capitalize on the opportunities presented by the integration of FinTech and eHealth. Firstly, healthcare institutions should prioritize investment in digital infrastructure and technology-enabled solutions to streamline administrative processes, enhance patient access to care, and improve overall operational efficiency. This may involve adopting integrated electronic health record systems, implementing secure digital payment platforms, and deploying telemedicine services to reach remote and underserved populations. Secondly, policymakers should establish clear regulatory frameworks and standards to govern the adoption and implementation of FinTech-enabled healthcare solutions, ensuring data privacy, security, and interoperability while fostering innovation and competition in the market. Thirdly, collaboration and partnerships between healthcare institutions, FinTech companies, academic institutions, and government agencies should be encouraged to drive research, development, and implementation of innovative FinTech-eHealth solutions tailored to the specific needs and challenges of the Saudi healthcare system. Additionally, efforts to promote digital literacy and technology adoption among healthcare professionals and patients should be prioritized to ensure equitable access and utilization of FinTech-enabled healthcare services across all segments of the population. By embracing these recommendations, Saudi Arabian healthcare institutions and policymakers can harness the transformative potential of FinTech and eHealth to improve healthcare delivery, enhance patient outcomes, and advance toward a more sustainable and inclusive healthcare system for all.

### 5.3 Limitations

Despite its contributions, this study has few limitations that should be acknowledged. Firstly, the qualitative nature of the research limits the generalizability of the findings to other contexts beyond Saudi Arabia, although may be used in the Middle East. Furthermore, the study focused solely on perspectives from Saudi Arabia, while potential insights from other regions or countries with different healthcare systems and regulatory environments were underscored.

## 6 Conclusion

In conclusion, this study has provided valuable insights into the interlinking of FinTech and eHealth in the context of Saudi Arabia, exploring the opportunities, challenges, and implications of integrating financial technology into healthcare delivery. Through qualitative interviews with healthcare professionals, FinTech consultants, and patients, the study has highlighted the significant potential of FinTech integration to enhance the efficiency, accessibility, affordability, and patient experience of healthcare services in the country. The findings underscore the importance of interdisciplinary collaboration and strategic planning in leveraging FinTech solutions to address longstanding challenges in the healthcare sector, such as administrative inefficiencies, access barriers, and data security concerns. However, the study also identifies several barriers, including regulatory complexities, interoperability issues, digital divide, resistance to change, and cost implications, which must be addressed to fully realize the benefits of FinTech integration in healthcare. Moving forward, policymakers, regulators, healthcare providers, technology developers, and other stakeholders must work together to overcome these barriers and foster an enabling environment for innovation and transformation in the Saudi healthcare ecosystem. By harnessing the transformative power of FinTech and eHealth, Saudi Arabia can enhance healthcare delivery, improve patient outcomes, and advance toward its vision of a sustainable and inclusive healthcare system for all.

## Data availability statement

The raw data supporting the conclusions of this article will be made available by the authors, without undue reservation.

## Author contributions

FA-A: Writing – original draft, Writing – review & editing.

## References

[1] Puschmann T. FinTech. Business & information. Syst Eng. (2017) 59:69–76. doi: 10.1007/s12599-017-0464-6

[2] Schueffel P. Taming the beast: a scientific definition of FinTech. J Innov Manag. (2017) 4:32–54. doi: 10.24840/2183-0606_004.004_0004

[3] Goldstein I Jiang W Karolyi GA. To FinTech and beyond. Rev Financ Stud. (2019) 32:1647–61. doi: 10.1093/rfs/hhz025

[4] Penedo FJ Oswald LB Kronenfeld JP Garcia SF Cella D Yanez B. The increasing value of eHealth in the delivery of patient-centered cancer care. Lancet Oncol. (2020) 21:e240–51. doi: 10.1016/s1470-2045(20)30021-8, PMID: 32359500 PMC7643123

[5] Uribe-Toril J Ruiz-Real JL Nievas-Soriano BJ. A study of eHealth from the perspective of social sciences. Healthcare. (2021) 9:108. doi: 10.3390/healthcare9020108, PMID: 33494182 PMC7909835

[6] Aceto G Persico V Pescapé A. Industry 4.0 and health: internet of things, big data, and cloud computing for healthcare 4.0. Journal of industrial information. Integration. (2020) 18:100129. doi: 10.1016/j.jii.2020.100129

[7] Klein A. Can FinTech improve health? Brookings education. (2021); Available at: https://www.brookings.edu/wp-content/uploads/2021/09/20210922_Klein_Can_fintech_improve_health.pdf

[8] Meiling L Yahya F Waqas M Shaohua Z Ali SA Hania A. Boosting sustainability in healthcare sector through FinTech: analyzing the moderating role of financial and ICT development. Inquiry. (2021) 58:004695802110281. doi: 10.1177/00469580211028174, PMID: 34167365 PMC8239971

[9] Arif LF Nurlaila Husein I Susilawati Nasution MLI Elveny M. Enhancement model for hospital quality service with consideration of integrating patients health insurance to utilize FINTECH. Sys Rev Pharm. (2020) 11:867.

[10] Singh S Sahni MM Kovid RK. What drives FinTech adoption? A multi-method evaluation using an adapted technology acceptance model. Manag Decis. (2020) 58:1675–97. doi: 10.1108/md-09-2019-1318

[11] Ahmad S Urus ST Nazri SNFSM. Technology acceptance of financial technology (FinTech) for payment services among employed fresh graduates. Asia Pacific Manag Account J. (2021) 16:27–58. doi: 10.24191/APMAJ.V16i2-02

[12] Al Nawayseh MK. FinTech in COVID-19 and beyond: what factors are affecting customers’ choice of FinTech applications? J Open Innov: Technol Mark Complex. (2020) 6:153. doi: 10.3390/joitmc6040153

[13] Yan C Siddik AB Akter N Dong Q. Factors influencing the adoption intention of using mobile financial service during the COVID-19 pandemic: the role of FinTech. Environ Sci Pollut Res. (2021) 30:61271–89. doi: 10.1007/s11356-021-17437-y, PMID: 34773583 PMC8589635

[14] Setiawan B Nugraha DP Irawan A Nathan RJ Zoltan Z. User innovativeness and FinTech adoption in Indonesia. J Open Innov: Technol Mark Complex. (2021) 7:188. doi: 10.3390/joitmc7030188

[15] Fosso Wamba S Kala Kamdjoug JR Epie Bawack R Keogh JG. Bitcoin, Blockchain and FinTech: a systematic review and case studies in the supply chain. Prod Plan Control. (2019) 31:115–42. doi: 10.1080/09537287.2019.1631460

[16] Kumari A Devi C. The impact of FinTech and Blockchain technologies on banking and financial services. Technol Innov Manag Rev. (2022) 12:1–11. doi: 10.22215/timreview/1481

[17] Golinelli D Boetto E Carullo G Nuzzolese AG Landini MP Fantini MP. Adoption of digital Technologies in Health Care during the COVID-19 pandemic: systematic review of early scientific literature. J Med Internet Res. (2020) 22:e22280. doi: 10.2196/22280, PMID: 33079693 PMC7652596

[18] Qadri YA Nauman A Zikria YB Vasilakos AV Kim SW. The future of healthcare internet of things: a survey of emerging technologies. IEEE Commun Surv Tutor. (2020) 22:1121–67. doi: 10.1109/comst.2020.2973314

[19] Rong G Mendez A Bou Assi E Zhao B Sawan M. Artificial Intelligence in healthcare: review and prediction case studies. Engineering. (2020) 6:291–301. doi: 10.1016/j.eng.2019.08.015

[20] Saraswat D Bhattacharya P Verma A Prasad VK Tanwar S Sharma G. Explainable AI for healthcare 5.0: opportunities and challenges. IEEE Access. (2022) 10:84486–517. doi: 10.1109/access.2022.3197671

[21] Secinaro S Calandra D Secinaro A Muthurangu V Biancone P. The role of artificial intelligence in healthcare: a structured literature review. BMC Med Inform Decis Mak. (2021) 21:125. doi: 10.1186/s12911-021-01488-9, PMID: 33836752 PMC8035061

[22] Zhang X Zhao T Wang L Dong Z. Does FinTech benefit financial disintermediation? Evidence based on provinces in China from 2013 to 2018. J Asian Econ. (2022) 82:101516. doi: 10.1016/j.asieco.2022.101516

[23] Urumsah D Ispridevi RF Nurherwening A Hardinto W. FinTech adoption: its determinants and organizational benefits in Indonesia. Jurnal Akuntansi Aud Indonesia. (2022) 88–101. doi: 10.20885/jaai.vol26.iss1.art9

[24] Delina LL. FinTech RE in a global finance Centre: expert perceptions of the benefits of and challenges to digital financing of distributed and decentralised renewables in Hong Kong. Energy Res Soc Sci. (2023) 97:102997. doi: 10.1016/j.erss.2023.102997

[25] Mugo C Njuguna A. Enhancing growth and productivity through mobile money financial technology services: the case of m-pesa in kenya. Inter J Eco Fin. (2023) 15:91. doi: 10.5539/ijef.v15n12p91

[26] Marwa S. Westgard E Khan F.. Can a FINTECH combination of BLOCKCHAIN, m-PESA and SMART contracts improve development project execution in sub SAHARAN Africa? (2018). Available at: https://scholar.google.com/citations?view_op=view_citation&hl=en&user=tnqDr9oAAAAJ&citation_for_view=tnqDr9oAAAAJ:u5HHmVD_uO8C (Accessed: 16 June 2024).

[27] Numi A Okemwa J Kingiri A. Reflections on FinTech and Covid-19: lessons from Kenya In: Singh L Joseph KJ, editors. Reimagining innovation systems in the COVID and post-COVID world. India: Routledge, (2023).

[28] Heston TF. A case study in Blockchain healthcare innovation Authorea (2017) (working paper).

[29] Soares B Ferreira A Veiga PM. The benefits and challenges of Blockchain technology and eHealth implementation in Estonia – a literature review. Appl Med Inform. (2023) 45:118–131.

[30] Rutschman Santos, Ana, healthcare Blockchain infrastructure: a comparative approach (2018). In Saint Louis U. Legal studies research paper no. 2018–4, Available at: https://ssrn.com/abstract=3217297

[31] Alalwan AA Baabdullah AM Al-Debei MM Raman R Alhitmi HK Abu-ElSamen AA. FinTech and contactless payment: help or hindrance? The role of invasion of privacy and information disclosure. Int J Bank Mark. (2023) 42:66–93. doi: 10.1108/ijbm-08-2022-0339

[32] Imam T McInnes A Colombage S Grose R. Opportunities and barriers for FinTech in SAARC and ASEAN countries. J Risk Finan Manag. (2022) 15:77. doi: 10.3390/jrfm15020077

[33] Yang K. Trust as an entry barrier: evidence from FinTech adoption. SSRN Electron J. (2021) 1–91. doi: 10.2139/ssrn.3761468

[34] Shala A Perri SR. Regulatory barriers for FinTech companies in central and Eastern Europe. Eastern J Eur Stud. (2022) 13:292–316. doi: 10.47743/ejes-2022-0214

[35] Haridh I. Language barriers as a limitation to achieving financial inclusion through FinTech in India. J Stud Res. (2022) 11:11. doi: 10.47611/jsrhs.v11i4.3198

[36] Odei-Appiah S Wiredu G Adjei JK. FinTech use, digital divide and financial inclusion. Digit Policy Regul Gov. (2022) 24:435–48. doi: 10.1108/dprg-09-2021-0111

[37] Mhlanga D. Prospects and challenges of digital financial inclusion/FinTech innovation in the fourth industrial revolution. Palgrave Stud Impact Finan. (2022):163–82. doi: 10.1007/978-3-031-16687-7_9

[38] Scott A.P. FinTech: overview of financial regulators and recent policy approaches. CRS report, (2020). Available at: https://www.everycrsreport.com/files/20200428_R46333_19f1ed5392343333e617b3ff00f188e518b2e6bc.pdf

[39] Feyen E Natarajan H Saal M. FinTech and the future of finance World Bank Publications (2023) (online).

[40] Askari S Ghofrani A Taherdoost H. “Fintech-enabled telemedicine: global healthcare advancement”, in Exploring Global Fintech Advancement and Applications. eds. Taherdoost H Le N Madanchian M Farhaoui Y Hoboken IGI Global, (2024) 313–330.

[41] Akinola S Telukdarie A. Sustainable digital transformation in healthcare: advancing a digital vascular health innovation solution. Sustain For. (2023) 15:10417. doi: 10.3390/su151310417

[42] Maher M Khan I Prikshat V. Monetisation of digital health data through a GDPR-compliant and blockchain enabled digital health data marketplace: a proposal to enhance patient’s engagement with health data repositories. Int J Inf Manag Data Insights. (2023) 3:100159. doi: 10.1016/j.jjimei.2023.100159

[43] Bonsu E. M. Baffour-Koduah D. From the Consumers’ Side: Determining Students’ Perception and Intention to Use ChatGPTin Ghanaian Higher Education. SSRN Elec J. (2023). doi: 10.2139/ssrn.4387107

[44] Kim H Sefcik JS Bradway C. Characteristics of qualitative descriptive studies: a systematic review. Res Nurs Health. (2017) 40:23–42. doi: 10.1002/nur.21768, PMID: 27686751 PMC5225027

[45] Braun V Clarke V. Using thematic analysis in psychology. Qual Res Psychol. (2006) 3:77–101. doi: 10.1191/1478088706qp063oa

[46] Statista. FinTech – Saudi Arabia. (2024); Available at: https://www.statista.com/outlook/dmo/fintech/saudi-arabia

[47] Intelligence Mordor. Market size of Saudi Arabia FinTech industry. (2024); Available at: https://www.mordorintelligence.com/industry-reports/saudi-arabia-fintech-market/market-size

[48] Intelligence Mordor. FinTech market size & share analysis – growth trends & forecasts. (2024); Available at: https://www.mordorintelligence.com/industry-reports/global-fintech-market

[49] Statista. Digital Payments – Saudi Arabia. (2024); Available at: https://www.statista.com/outlook/dmo/fintech/digital-payments/saudi-arabia

[50] Statista. Digital payments – worldwide. (2024); Available at: https://www.statista.com/outlook/dmo/fintech/digital-payments/worldwide

[51] Albarrak MS Alokley SA. FinTech: ecosystem, opportunities and challenges in Saudi Arabia. J Risk Finan Manag. (2021) 14:460. doi: 10.3390/jrfm14100460

[52] Khan S Al-Harby ASA. The use of FINTECH and its impact on financial intermediation – a comparison of Saudi Arabia with other GCC economies. Intellect Econ. (2022) 16:26–43.

[53] Oladapo IA Hamoudah MM Alam MM Olaopa OR Muda R. Customers’ perceptions of FinTech adaptability in the Islamic banking sector: comparative study on Malaysia and Saudi Arabia. J Model Manag. (2021) 17:1241–61. doi: 10.1108/jm2-10-2020-0256

[54] Cambaza E. The role of FinTech in sustainable healthcare development in sub-Saharan Africa: a narrative review. FinTech. (2023) 2:444–60. doi: 10.3390/fintech2030025

[55] Inggrit WL Zunairoh Z Andri R. Does financial technology matter? Evidence from the Indonesia healthcare industry during the Covid-19 pandemic. SciPap. (2022) 30:1509. doi: 10.46585/sp30011509

